# Splenic uptake on FDG PET/CT correlates with Kikuchi-Fujimoto disease severity

**DOI:** 10.1038/s41598-021-90350-z

**Published:** 2021-05-25

**Authors:** Hye Seong, Yong Hyu Jeong, Woon Ji Lee, Jun Hyoung Kim, Jung Ho Kim, Jin Young Ahn, Su Jin Jeong, Jun Yong Choi, Yoon Soo Park, Joon Sup Yeom, Young Goo Song, Arthur Cho, Nam Su Ku

**Affiliations:** 1 Department of Internal Medicine and AIDS Research Institute, Yonsei University College of Medicine, 50-1 Yonsei-ro, Seodaemun-gu, Seoul, 120-752 Republic of Korea; 2 Department of Nuclear Medicine, Severance Hospital, Yonsei University College of Medicine, 50-1 Yonsei-ro, Seodaemun-gu, Seoul, 120-752 Republic of Korea; 3 Department of Internal Medicine, Korea University College of Medicine, Seoul, Republic of Korea

**Keywords:** Diseases, Medical research

## Abstract

Kikuchi-Fujimoto disease (KFD) is usually self-limiting, but prolonged systemic symptoms often result in frequent hospital visits, long admission durations, or missed workdays. We investigated the role of fluorine-18 fluoro-2-deoxy-D-glucose (^18^F-FDG) positron emission tomography/computed tomography (PET/CT) in assessing KFD severity. We reviewed the records of 31 adult patients with pathologically confirmed KFD who underwent ^18^F-FDG PET/CT between November 2007 and April 2018 at a tertiary-care referral hospital. Disease severity was assessed using criteria based on clinical manifestations of advanced KFD. Systemic activated lymph nodes and severity of splenic activation were determined using semi-quantitative and volumetric PET/CT parameters. The median of the mean splenic standardized uptake value (SUV_mean_) was higher in patients with severe KFD than those with mild KFD (2.38 ± 1.18 vs. 1.79 ± 0.99, *p* = 0.058). Patients with severe KFD had more systemically activated volume and glycolytic activity than those with mild KFD (total lesion glycolysis: 473.5 ± 504.4 vs. 201.6 ± 363.5, *p* = 0.024). Multivariate logistic regression showed that myalgia (odds ratio [OR] 0.035; 95% confidence interval [CI] 0.001–0.792; *p* = 0.035), total lymph node SUV_max_ (cutoff 9.27; OR 24.734; 95% CI 1.323–462.407; *p* = 0.032), and spleen SUV_mean_ (cutoff 1.79; OR 37.770; 95% CI 1.769–806.583; *p* = 0.020) were significantly associated with severe KFD. ^18^F-FDG PET/CT could be useful in assessing KFD severity.

## Introduction

Kikuchi-Fujimoto disease (KFD), also known as histiocytic necrotizing lymphadenitis, is a disease endemic to Asia and of unknown etiology^1–3^. It usually develops in young adult women and is most commonly characterized by cervical lymphadenopathy and fever^4,5^. KFD presents with various clinical features, ranging from absence of systemic symptoms to significant symptoms like night sweats, myalgia, weight loss, arthralgia, and hemophagocytic lymphohistiocytosis (HLH)^6–9^.

Although KFD is usually benign and self-limiting, patients with prolonged systemic symptoms are plagued with frequent hospital visits, long durations of admission, or missed workdays^10^. Immunomodulating drugs, such as high-dose corticosteroids or intravenous immunoglobulins, which aid in shortening the clinical course of the disease, have been administered for treating patients with severe KFD^11,12^. However, as there are no established markers for KFD severity, the determination of treatment options for KFD is dependent only on the clinician’s discretion, which may result in delayed treatment or a prolonged symptom duration.

Fluorine-18 fluoro-2-deoxy-d-glucose (^18^F-FDG) positron emission tomography/computed tomography (PET/CT) can be used to evaluate glucose utilization in multiple organs. Although ^18^F-FDG uptake has been predominately used to evaluate cancer metabolism, ^18^F-FDG PET/CT is used in clinical settings to assess localized inflammatory foci and infectious diseases, such as tuberculosis, Q fever, infective endocarditis, vascular graft infection, chronic active Epstein–Barr virus infection, invasive fungal infection, and surgical site infection^13–17^. Another advantage of ^18^F-FDG PET/CT is that PET metrics, such as standardized uptake value (SUV), metabolic tumor volume (MTV), and total lesion glycolysis (TLG), allow for the evaluation of the severity and quantification of glycolysis in multiple organs, which serve as prognostic prediction parameters for survival in patients with solid tumors^18^.

The spleen is an important immune organ in both innate and adaptive immune responses and in regulating immune homeostasis^19^. Studies have reported diffuse increased splenic ^18^F-FDG uptake in patients with lymphoma, infections, tuberculosis, and autoimmune diseases^20–23^. Moreover, recent findings suggest that a diffuse increased FDG uptake was observed not only in the lymph nodes but also in the spleen in patients with KFD^24,25^. However, the relationship between abnormal ^18^F-FDG uptake in patients with KFD and disease severity has not been established to date.

We hypothesized that glucose metabolism in the spleen and pathologic lymph nodes varies according to KFD severity because of the presence of systemic inflammation. Hence, we aimed to investigate the ^18^F-FDG uptake in the spleen and lymph nodes in patients with KFD and evaluate its performance as a disease severity parameter.

## Methods

### Patient selection

#### Inclusion criteria

We retrospectively reviewed the electronic medical records between November 2007 and April 2018 at a tertiary-care referral hospital located in Seoul, Korea. We enrolled patients who had pathologically confirmed KFD and underwent ^18^F-FDG PET/CT in the same admission period.

#### Exclusion criteria

We excluded patients who did not meet the criteria to assess KFD severity.

#### Data collections

Data collected included age, sex, clinical manifestations, laboratory test values, histologic findings, treatment methods and durations, fever duration, and outcomes such as relapse. All procedures performed in human studies were in accordance with the ethical standards of the institutional and/or national research committee and with the 1964 Helsinki Declaration and its later amendments or comparable ethical standards. This study was approved by the Institutional Review Board of Yonsei University Health System Clinical Trial Center (4-2019-0977). Since the study was retrospective and the study subjects were anonymized, the Institutional Review Board Review Board of Yonsei University Health System Clinical Trial Center waived the requirement for written consent from the patients.

### Definition of severe KFD

For assessment of disease severity, we established the presence of severe KFD based on the clinical manifestations of advanced KFD disease^10,26–28^. Severe KFD was defined as KFD with at least one of the following manifestations: encephalitis, peripheral neuropathy, HLH, long fever duration (> 7 days), or leukopenia (< 4000/μL).

### Imaging technique

All patients fasted for at least 6 h before the PET/CT scans were taken. Serum glucose levels (preferably < 150 mg/dL) were measured followed by intravenous administration of 5.5 MBq/kg ^18^F-FDG (with a maximum of 400 MBq). PET and combined low-dose CT scans were performed with commercial PET/CT scanners (Discovery STE, Discovery D600, Discovery D710 [GE Healthcare, Chicago, IL, USA], or Biograph TruePoint40 [Siemens Healthineers, Erlangen, Germany]) after 1 h. The PET scan was performed with an acquisition time of 2 min per bed position in the 3-dimensional mode. PET data were reconstructed iteratively using an ordered subset expectation maximization algorithm with the low-dose CT datasets for attenuation correction.

### Imaging analysis

**Figure 1:**
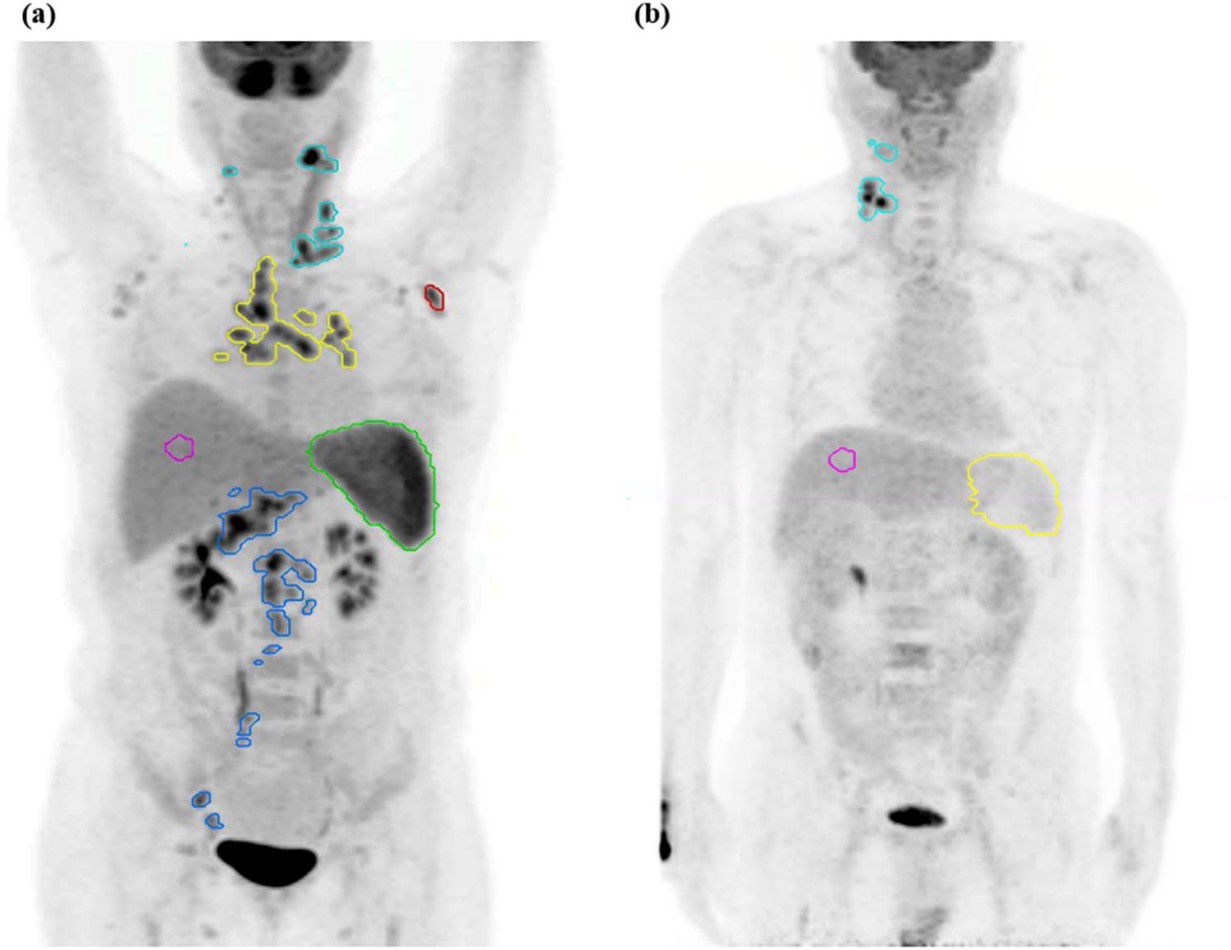
Maximum intensity projection images of representative mild and severe Kikuchi-Fujimoto disease patients with lesion segmentation. (**a**) A patient with severe Kikuchi-Fujimoto disease. Systemic increase in ^18^F-FDG uptake in the spleen and lymph nodes is seen. (**b**) A patient with mild Kikuchi-Fujimoto disease. Lesser FDG-avid lymph nodes are seen.

For semi-quantitative and volumetric analysis, various metabolic PET parameters were measured including the maximum SUV (SUV_max_), MTV, and TLG in the lymph nodes as well as the mean SUV (SUV_mean_) and TLG in the spleen using commercially available imaging software (MIM Software, Cleveland, OH, USA). All PET/CT images were read by two experienced nuclear medicine physicians. For background activity, a spherical volume of interest (VOI) with a diameter of 30 mm was drawn at the inferior right lobe of the liver, excluding the main ducts and vessels. The SUV_mean_ of that VOI was adopted as a threshold value to determine the boundaries of the pathologic lymph nodes in each PET/CT study. After SUV thresholding, FDG-avid regions were automatically segmented using the isocontour threshold method. The SUV_max_ of the total lymph nodes was defined as the highest metabolic foci (SUV_max_) in all the metabolically active lymph nodes in the body. The total MTV of lymph nodes was defined as the sum of the MTVs of all individual focal lesions identified in the analysis. The TLG of each focal lesion was calculated by multiplying the SUV_mean_ and voxel number of that lesion. The total lymph node TLG of each patient was defined as the sum of the TLGs for all focal lesions in the analysis. The SUV_mean_ and TLG of the spleen were identified by manually drawing regions of interest on each slice of the attenuation-corrected axial PET images (Fig. 1).

### Statistical analysis

All our statistical analysis were only two variables (mild, severe). Descriptive statistics for continuous variables are presented as medians ± interquartile range (IQR), and categorical variables are shown as numbers (percentage). The Mann–Whitney *U* test was performed to analyze differences between the mild and severe groups, while the *χ*^2^ test or Fisher’s exact test was performed on categorical data using SPSS 25.0 (IBM, Armonk, NY, USA). To determine independent predictors of severity in the KFD group, we performed a multivariable analysis with a logistic regression model including risk factors associated with a *p* value of less than 0.05 in the univariate analysis. Differences were considered to be statistically significant at a 2-sided *p* value of less than 0.05.

Receiver operating characteristic (ROC) analysis was used to describe the relationship between FDG uptake and disease severity. MedCalc software (version 19.1, Ostend, Belgium) was used to analyze the data. Results of ^18^F-FDG PET/CT in the severe group were compared with those in the mild group to assess the diagnostic performance of ^18^F-FDG PET/CT in evaluating the degree of severity of KFD. The diagnostic performance was expressed in terms of sensitivity, specificity, Youden index, positive predictive value (PPV), and negative predictive value (NPV).

## Results

### Patient characteristics

The baseline characteristics of the 31 patients with KFD who underwent ^18^F-FDG PET/CT are divided into two groups—mild and severe KFD—and summarized in Table 1. The *p* values shown are the result from just two variables (mild, severe). All patients had been confirmed with KFD based on the pathological findings of a biopsy. The median age was 27.5 years (IQR, 28 years), and 13 patients were male (41.9%). The most commonly affected site of lymphadenopathy was the neck (n = 18; 58.1%), followed by the axilla (n = 7; 22.6%). In terms of systemic symptoms, almost every patient in our study presented with a fever (n = 30; 96.8%). Among the study patients, 8 (25.8%) patients were categorized into the mild and 23 (74.2%) into the severe KFD group. There were significant differences in age (38.0 ± 29 vs. 26.0 ± 18 years, *p* = 0.038) and lactate dehydrogenase (LDH) levels (310.5 ± 298 vs. 612.5 ± 672, *p* = 0.033). However, there were no significant differences in sex (57.1% vs. 37.5%, *p* = 0.354), sites of lymphadenopathy, or systemic symptoms between the mild and severe groups.

**Table 1:** Patient characteristics and results of univariate analysis for predicting the severity of Kikuchi-Fujimoto disease.

Characteristics	Total (*n* = 31)	Mild (*n* = 8)*	Severe (*n* = 23)*	*p* value*
Age (years)	27.5 ± 28	38.0 ± 29	26.0 ± 18	0.038
Sex (male, %)	13 (41.9)	3 (37.5)	10 (43.5)	> 0.999
**Lymphadenopathy, yes (%)**				
Cervical	18 (58.1)	5 (62.5)	13 (56.5)	> 0.999
Axillary	7 (22.6)	2 (25.0)	5 (21.7)	> 0.999
Mediastinal	2 (6.5)	1 (12.5)	1 (4.3)	0.456
Abdominopelvic	4 (12.9)	0 (0.0)	4 (17.4)	0.550
**Systemic symptoms**				
Fever	30 (96.8)	8 (100.0)	22 (95.7)	> 0.999
Night sweat	7 (22.6)	2 (25.0)	5 (21.7)	> 0.999
Sore throat	4 (12.9)	2 (25.0)	2 (8.7)	0.268
Weight loss	5 (16.1)	2 (25.0)	3 (13.0)	0.583
Rash	8 (25.8)	3 (37.5)	5 (21.7)	0.393
Myalgia	7 (22.6)	4 (57.1)	3 (42.9)	0.053
Arthralgia	5 (16.1)	3 (37.5)	2 (8.7)	0.093
Nausea	4 (12.9)	1 (12.5)	3 (13.0)	> 0.999
Headache	2 (6.5)	1 (12.5)	1 (4.3)	0.456
**Laboratory findings**				
WBC (10^3^/ul)	3.720 ± 4.52	6.645 ± 3.64	2.595 ± 1.97	0.002
CRP (mg/L)	22.75 ± 73.35	71.93 ± 107.50	15.9 ± 51.71	0.091
ESR (mm/h)	48.0 ± 39.75	67.0 ± 40.5	47.5 ± 43.5	0.348
LDH (IU/L)	505.5 ± 604	310.5 ± 298	612.5 ± 672	0.033
**PET/CT parameters**				
Total lymph nodes SUV_max_	10.65 ± 7.64	8.19 ± 7.10	11.68 ± 7.33	0.214
Total lymph nodes MTV	97.95 ± 112.29	61.85 ± 118.32	99.36 ± 120.21	0.104
Total lymph nodes TLG	398.73 ± 464.08	201.57 ± 363.45	473.52 ± 504.44	0.024
Spleen SUV_mean_	2.19 ± 1.14	1.79 ± 0.99	2.38 ± 1.18	0.058
Spleen TLG	539.24 ± 395.69	391.78 ± 528.87	548.73 ± 380.91	0.162

WBC, white blood cell count; CRP, C-reactive protein; ESR, erythrocyte sedimentation rate; LDH, lactate dehydrogenase; PET/CT, positron emission tomography/computed tomography; SUV_max_, maximum standardized uptake value; MTV, metabolic tumor volume; TLG, total lesion glycolysis; SUV_mean_, mean standardized uptake value

Continuous variables are presented as means ± standard deviation and medians ± interquartile range (3rd interquartile range-1st interquartile range), and categorical variables are presented as numbers (percentage)

*Statistical analysis performed mild and severe KFD.

### Comparison of PET/CT parameters according to the severity of KFD

We investigated the locations, metabolic activity, and size of hypermetabolic lymph nodes on ^18^F-FDG PET/CT images. The findings from the ^18^F-FDG PET/CT examinations are presented in Table 1. We identified hypermetabolic lymph nodes in 31 patients with SUV_max_ values from the neck, axilla, mediastinum, and the abdominopelvic area. Hypermetabolic lymph nodes were observed in the necks of 18 patients, axillae of 7, mediastina of 4, and abdomens and pelvis of two patients. The median values of SUV_max_, MTV, and TLG of ^18^F-FDG uptake in affected lymph nodes were 10.65 ± 7.64, 97.95 ± 112.29, and 398.73 ± 464.08, respectively. The ^18^F-FDG uptake in the spleen (SUV_mean_, 2.19 ± 1.14; TLG, 539.36 ± 395.69) was calculated for all patients.

The median ^18^F-FDG PET/CT parameters involving the lymph nodes, liver, and spleen were identified in the mild and severe groups. The spleen SUV_mean_ was higher in patients with severe KFD (1.79 ± 0.99 vs. 2.38 ± 1.18, *p* = 0.058). The median values of total lymph node SUV_max_ (8.19 ± 7.10 vs. 11.68 ± 7.33, *p* = 0.214), MTV (61.85 ± 118.32 vs. 99.36 ± 120.21, *p* = 0.104), and total lymph node TLG (201.57 ± 363.45 vs. 473.52 ± 504.44, *p* = 0.024) were higher in the severe group than in the mild group, indicating a higher ^18^F-FDG uptake in the severe group.

### Diagnostic performance of ^18^F-FDG PET/CT in the prediction of KFD severity

**Figure 2:**
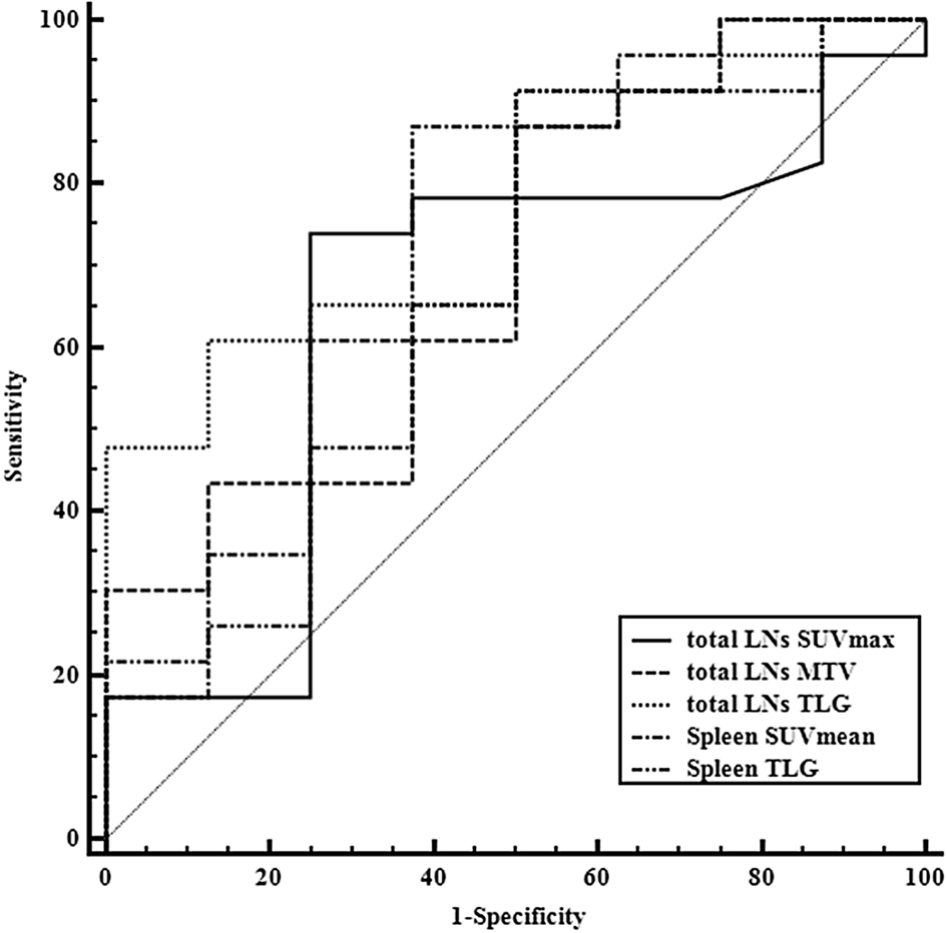
Receiver operating characteristic curve of metabolic parameters in Kikuchi-Fujimoto disease. The areas under the curve (AUC) for total lymph nodes, TLG, and spleen SUV_mean_ are higher than that of total lymph nodes MTV. SUV_mean_, mean standardized uptake value; MTV, metabolic tumor volume; TLG, total lesion glycolysis.

Severity predictions were made by analyzing the area under the curve (AUC) of the ROC (Fig. 2), and the corresponding statistics are shown in Table 2. Using the definition of severe KFD as a diagnostic criterion to separate the severe group from the mild group, ROC curve analysis determined the most sensitive and specific cutoff values for total lymph node TLG, spleen SUV_mean_, total lymph node MTV, spleen TLG, and total lymph node SUV_max_ as 429.99, 1.79, 34.72, 296.06, and 9.27, respectively. With these cutoff values, total lymph node TLG (77.2%) and spleen SUV_mean_ (72.8%) were found to be more accurate than the other parameters.

**Table 2:** Diagnostic performance of positron emission tomography/computed tomography for the severity of Kikuchi-Fujimoto disease.

Parameters	Cutoff	Sensitivity (%)	Specificity (%)	Accuracy (%)	Youden index	PPV (%)	NPV (%)
Total lymph nodes SUV_max_	9.27	73.9	75.0	64.9	0.4891	89.5	50.0
Total lymph nodes MTV	34.72	87.0	50.0	69.6	0.3696	83.3	57.1
Total lymph nodes TLG	429.99	60.9	87.5	77.2	0.4837	93.3	43.7
Spleen SUV_mean_	1.79	87.0	62.5	72.8	0.4946	87.0	62.5
Spleen TLG	296.06	91.3	50.0	66.8	0.4130	84.0	66.7

PPV, positive predictive value; NPV, negative predictive value; SUV_max_, maximum standardized uptake value; MTV, metabolic tumor volume; TLG, total lesion glycolysis; SUV_mean_, mean standardized uptake value

### Predictive factors for KFD severity

We evaluated the predictive factors for severe KFD. A univariate and multivariable analysis of severity in KFD was performed and is presented in Table 3 and Table S1. In the univariate logistic regression, myalgia (odds ratio [OR] 0.150; 95% confidence interval [CI] 0.024–0.946; *p* = 0.044), total lymph node SUV_max_ (cutoff 9.27) (OR 8.500; 95% CI 1.335–54.127; *p* = 0.023), total lymph node MTV (cutoff 34.72) (OR 6.667; 95% CI 1.057–42.065; *p* = 0.044), total lymph node TLG (cutoff 429.99) (OR 10.889; 95% CI 1.140–103.977; *p* = 0.038), spleen SUV_mean_ (cutoff 1.79) (OR 11.111; 95% CI 1.701–72.564; *p* = 0.012), and spleen TLG (cutoff 296.06) (OR 10.500; 95% CI 1.412–78.059; *p* = 0.022) were statistically significant. The multivariate logistic regression model showed that myalgia (OR 0.035; 95% CI 0.001–0.792; *p* = 0.035), total lymph node SUV_max_ (cutoff 9.27) (OR 24.734; 95% CI 1.323–462.407; *p* = 0.032), and spleen SUV_mean_ (cutoff 1.79) (OR 37.770; 95% CI 1.769–806.583; *p* = 0.020) were significantly associated with severe KFD.

**Table 3:** Factors associated with severe Kikuchi-Fujimoto disease using logistic regression model.

Variables	Univariate		Multivariable	
	OR (95% CI)	*P* value	OR (95% CI)	*P* value
Age	0.956 (0.910–1.004)	0.073		
**Sex**		0.768		
Female	Reference			
Male	1.282 (0.246–6.688)			
**Systemic symptoms**				
Fever duration	1.212 (0.941–1.560)	0.137		
Myalgia	0.150 (0.024–0.946)	0.044	0.035 (0.001–0.792)	0.035
**Laboratory findings**				
CRP (≥ 100 mg/L)	0.632 (0.092–4.350)	0.641		
LDH (≥ 1000 IU/L)	1.944 (0.192–19.741)	0.574		
**PET-CT parameters**				
Total lymph nodes SUV_max_ (> 9.27)	8.500 (1.335–54.127)	0.023	24.734 (1.323–462.407)	0.032
Total lymph nodes MTV (> 34.72)	6.667 (1.057–42.065)	0.044		
Total lymph nodes TLG (> 429.99)	10.889 (1.140–103.977)	0.038		
Spleen SUV_mean_ (> 1.79)	11.111 (1.701–72.564)	0.012	37.770 (1.769–806.583)	0.020
Spleen TLG (> 296.06)	10.500 (1.412–78.059)	0.022		
Relapse	1.050 (0.093–11.824)	0.968		
Steroid use	0.762 (0.122–4.751)	0.771		

OR, Odds ratio; CI, confidential interval; SUV_max_, maximum standardized uptake value; SUV_mean_, mean standardized uptake value; MTV, metabolic tumor volume; TLG, total lesion glycolysis; CRP, C-reactive protein; LDH, lactate dehydrogenase

## Discussion

^18^F-FDG PET/CT can be used to investigate various inflammatory and infectious diseases and benign disorders^29^. Due to the advantages of ^18^F-FDG PET/CT in the systematic evaluation of fever of unknown origin^30^, ^18^F-FDG uptake has often been assessed in the diagnostic workup of KFD. Alshammari et al. reported that ^18^F-FDG uptake can be detected not only in the generalized lymph nodes but also in the spleen in patients with KFD^24^. Another study reported that the spleen showed increased ^18^F-FDG uptake in patients with febrile autoimmune disease and is associated with an increased risk of all-cause in-hospital mortality^22^.

In this study, myalgia was found to be correlated with mild KFD. This may be because patients with mild KFD often present with myalgia at the time of diagnosis. Furthermore, patients presenting with myalgia as a systemic symptom are usually evaluated for the disease earlier than those who do not present with myalgia. We investigated the values of ^18^F-FDG PET/CT in patients with severe KFD to determine whether they can be used as predictive factors for disease severity. Among the various ^18^F-FDG PET/CT parameters, total lymph node SUV_max_ and spleen SUV_mean_ were significantly associated with severe KFD. ^18^F-FDG uptake was significantly higher not only in the affected lymph nodes but also in the spleen in severe KFD. In multivariate logistic regression analysis, total lymph node SUV_max_ with a cutoff value higher than 9.27 and spleen SUV_mean_ with a cutoff value higher than 1.79 were independent predictors of KFD severity. Increased total lymph node SUV_max_ and spleen SUV_mean_ might be useful for predicting the disease course when clinical or laboratory data are not available or are not confirmed. We have shown, using multiple multivariable models, that not only the intensity of inflammatory response in lymph nodes (SUV_max_), but also the amount of activated lymph nodes (MTV, TLG) is correlated with KFD severity. Similarly, we have shown that spleen intensity (SUV_max_) as well as splenic metabolic size (MTV) is also correlated with KFD severity.

The spleen is the largest lymphoid organ in the human body that regulates blood flow and filters microorganisms^19^. As a specialized immune organ, the spleen has various functions, such as clearance of microorganisms, the site of development for lymphocytes (both T and B), release of immunoglobulins, and production of immune mediators^31^. Generally, ^18^F-FDG uptake is related to tissue metabolism, which may explain why an increased ^18^F-FDG uptake in the spleen may reflect increased glucose consumption in the spleen in the event of an infection^21^. A recent study demonstrated that current inflammation could result in diffuse splenic ^18^F-FDG uptake^32^. Therefore, we presume that increased diffuse ^18^F-FDG uptake in the spleen can be noted in many inflammatory diseases reflecting the activation of the immune system in the spleen. This relationship between splenic glucose metabolism and inflammation may help explain our results.

There are several limitations of this study. First, this was a retrospective study. Second, our study population was small due to the low prevalence of KFD and the high cost of ^18^F-FDG PET/CT. Finally, we defined severe KFD arbitrarily. Since the severity criteria of KFD have not been previously defined, we defined severe KFD based on previous reports assessed factors associated with a severe clinical course and fetal complications of KFD^3,10,26–28,33^ and our clinical experience. Despite these limitations, our study is the first, to the best of our knowledge, to evaluate the potential association between ^18^F-FDG PET/CT parameters and KFD severity.

Our study suggests that ^18^F-FDG PET/CT can be a useful tool to assess disease severity in patients with KFD as a complement to laboratory and clinical findings. Further studies with larger populations are warranted to validate our results regarding the role of ^18^F-FDG PET/CT in determining KFD severity.

## Supplementary Information

Supplementary Informations.

## Data Availability

The datasets used and/or analyzed during the current study are available from the corresponding author on reasonable request.
