# Supplementary material for: Splenic uptake on FDG PET/CT correlates with Kikuchi-Fujimoto disease severity
Source: Sci Rep. 2021 May 25;11:10836. doi: 10.1038/s41598-021-90350-z (PMC8149816; doi:10.1038/s41598-021-90350-z)
Supplement: Supplementary file 1 — Supplementary Informations. [file 41598_2021_90350_MOESM1_ESM.docx]

**Supplementary Appendix**

This appendix has been provided by the authors to give readers additional information about their work.

Supplement to Hye Seong, Yong Hyu Jeong, Woon Ji Lee, Jun Hyoung Kim, Jung Ho Kim, Jin Young Ahn, Su Jin Jeong, Jun Yong Choi, Yoon Soo Park, Joon Sup Yeom, Young Goo Song, Arthur Cho, and Nam Su Ku. Splenic uptake on FDG PET/CT correlates with Kikuchi-Fujimoto disease severity. Scientific Reports.

**Supplementary Appendix**

**Title:** Splenic uptake on FDG PET/CT correlates with Kikuchi-Fujimoto disease severity

**Table of contents**

Table S1. Four other multivariable logistic regression models using risk factors associated with severe Kikuchi-Fujimoto disease............................................................................. 3

| **Supplementary Table 1. Four other multivariable logistic regression models using risk factors associated with severe Kikuchi-Fujimoto disease** | | | | | | | | | | | | | | | | | | | | | | | | | |
| --- | --- | --- | --- | --- | --- | --- | --- | --- | --- | --- | --- | --- | --- | --- | --- | --- | --- | --- | --- | --- | --- | --- | --- | --- | --- |
| **Variables** | **Multivariable 1** | | | | **Multivariable 2** | | | | | **Multivariable 3** | | | | | | | | | | **Multivariable 4** | | | | | |
|  | **OR (95% CI)** | ***P*** | | **OR (95% CI)** | | ***P*** | | | **OR (95% CI)** | | | | | | | ***P*** | | | | **OR (95% CI)** | | | ***P*** | | |
| Myalgia | 0.052 (0.003-0.902) | 0.042 | | 0.041 (0.002-0.674) | | | 0.025 | | 0.000 (0.000-.) | | | | | | 0.998 | | | | 0.000 (0.000-.) | | | 0.998 | | |  |
| Total lymph nodes SUV_max_ (> 9.27) |  |  | |  | | | | |  | | | | | |  | | | |  | | | | | |  |
| Total lymph nodes MTV (> 34.72) | 8.832 (0.642-121.571) | 0.103 | | 6.394 (0.463-88.352) | | | 0.166 | | | | |  |  | | | | | | | |  | | | |  |
| Total lymph nodes TLG (> 429.99) |  |  | |  | | | | 1792807920 (0.000-.) 0.998 | | | | | | | | | | 962048059.4 (0.000-.) | | | | | | 0.998 |  |
| Spleen SUV_mean_ (> 1.79) | 22.465 (1.483-340.294) | 0.025 | | 20.412 (1.364-305.393) 0.029 | | | | | | | | | | | | | | | | | | | | |  |
| Spleen TLG (> 296.06) |  | | 25.423 (1.454-444.572) | | | | 0.027 | | | |  | | |  | | | 24.000 (1.111-518.581) 0.043 | | | | | | | |  |
| OR, odds ratio; CI, confidential interval; CRP, C-reactive protein; LDH, lactate dehydrogenase; PET/CT, positron emission tomography-computed tomography; SUV_max_, maximum standardized uptake value; MTV, metabolic tumor volume; TLG, total lesion glycolysis; SUV_mean_, mean standardized uptake value. | | | | | | | | | | | | | | | | | | | | | | | | | |
